# A porous ceramic particle with or without a preservative blend did not impair apparent digestibility of macro- and micro-nutrients of postweaned pigs

**DOI:** 10.1093/tas/txac078

**Published:** 2022-06-09

**Authors:** Emily M Davis, Yu Liang, Kayla P Wallace, Amanda J Zimmerman, Matthew G Siebecker, Paul Rand Broadway, Jeffrey A Carroll, Michael A Ballou

**Affiliations:** Department of Veterinary Sciences, Texas Tech University, Lubbock, TX 79409, USA; Department of Veterinary Sciences, Texas Tech University, Lubbock, TX 79409, USA; Texas Tech University School of Veterinary Medicine, Amarillo, TX 79106, USA; Department of Plant and Soil Sciences, Texas Tech University, Lubbock, TX 79409, USA; Department of Plant and Soil Sciences, Texas Tech University, Lubbock, TX 79409, USA; Livestock Issues Research Unit, Agricultural Research Service USDA, Lubbock, TX 79403, USA; Livestock Issues Research Unit, Agricultural Research Service USDA, Lubbock, TX 79403, USA; Department of Veterinary Sciences, Texas Tech University, Lubbock, TX 79409, USA

**Keywords:** ceramic, clay, micronutrient, montmorillonite

## Abstract

The objective of this study was to determine the effects of supplementing a commercial porous ceramic clay particle, with or without a blend of preservatives, on the performance and nutrient digestibility of weanling pigs. Fifteen weanling pigs of the Yorkshire, Landrace, and Duroc breeds were blocked by breed and randomly assigned to one of three treatments (*n* = 5): (1) Control, non-medicated diet with no additional feed additives (CON); (2) PowerGuard, basal diet with 0.25% of the DM consisting of a ceramic particle mixed into the pelleted feed (PG; MB Nutritional Sciences, Lubbock, TX, 79403); or (3) Power Guard + a blend of preservatives, basal diet with 0.3% of the DM consisting of the ceramic clay and preservatives mixed into the pelleted feed (PG-D). The facility was temperature controlled with an average temperature of 28.5 °C. Pigs were offered ad libitum access to feed and water and were housed individually in elevated crates. Body weights were collected upon enrollment on day 0 and at the end of the observation period on day 18. On day 15 , a 72-h total feed and fecal collection period began. Feed and fecal samples were analyzed for DM, CP, Ash, OM, ADF, NDF, zinc, copper, thiamin (vitamin B1), and retinol (vitamin A). Liver samples were collected immediately after harvest and frozen for later mineral analysis. Data were analyzed using Proc Mixed in SAS with dietary group as the main effect and block as the random effect (SAS 9.4, Cary, NC). There were no treatment differences in performance measures including final BW, ADG, or G:F (*P* ≥ 0.701). There were no treatment differences in diet nutrient digestibility for DM, CP, Ash, OM, ADF, or NDF (*P* ≥ 0.312). Additionally, there were no treatment effects on zinc, copper, or retinol digestibility (*P* ≥ .298); however, thiamin inclusion rate was increased for the PG-D treatment, thus leading to an increased digestibility for thiamin (*P* = 0.018) in the PG-D treatment. There were no treatment differences in hepatic mineral concentrations (*P* ≥ 0.532); however, there was a tendency for pigs fed PG-D to have increased hepatic concentrations of lead and mercury when compared with both PG and CON pigs (*P* ≤ 0.066). In summary, supplementation of a commercial ceramic particle with or without a blend of preservatives to weaned pigs did not affect performance or apparent nutrient digestibility.

## INTRODUCTION

Supplementing clay-based nutritional products to pigs to ameliorate the effects of various biotoxins, mainly mycotoxins and bacterial toxins, can be effective in reducing negative health and performance effects due to diet contamination. Due to the structural and biological difference among clays, each must be evaluated for the efficacy, safety, and potential nutrient binding capabilities both in vivo and in vitro before being introduced into feed to exert this potentially protective effect, especially if it will be fed for a prolonged period of time.

Clays have been supplemented to livestock species for decades with equivocal results, in part due to the different biological capabilities among clays (Emmerich et al., 2009; Song et al., 2012; Gouda, 2019). The classification system used for clays is broad and can be based on the interlayer structure, chemical formula, as well as mineral and interlayer contents, thus leaving some difficulty for comparisons when these basic features vary greatly among samples. The mineral deposit used in the current study is from the bentonite-smectite group, specifically a montmorillonite clay that was thermally processed. Thermal processing of clays can increase pore size, create micropores, and shift the interlayer structure to allow for increased surface area for adsorption capacity (Murray, 2000; Qu et al., 2018). The thermally processed clay in the current study will be referred to as ceramic particle due to the changes in physical structure that occur with this specific processing. When a clay is thermally processed into a ceramic the physical structure is permanently changed whereas the particle can no longer be re hydrated into a clay. With the increase in surface area leading to increased adsorption potential, ceramic particles have been shown to have broad-spectrum abilities from in vitro binding to biotoxins to increasing performance and health in vivo (Schell et al., 1993; Diaz et al., 2002; Jiang et al., 2010).

Given this current knowledge gap, the objective of this study was to evaluate the effects of a ceramic particle with or without a blend of preservatives on the performance and apparent nutrient digestibility of weanling pigs.

## MATERIALS AND METHODS

All procedures in this study were approved by the USDA-ARS, Livestock Issues Research Unit’s Institutional Animal Care and Use Committee (IACUC protocol #LIRU-2017F).

### Study Design

Fifteen weanling pigs of the Yorkshire, Landrace, and Duroc breeds were utilized in the current study, blocked by breed and randomly assigned to one of three treatments (*n* = 5): (1) Control, non-medicated diet with no feed additives (CON); (2) PowerGuard, basal diet with 0.25% of the DM consisting of a ceramic particle mixed with the pelleted feed (PG; MB Nutritional Sciences, Lubbock, TX, 79403); or (3) PowerGuard + a blend of preservatives (sodium metabisulfite (SMB) and ethoxyquin) + thiamin, basal diet with 0.3% of the DM consisting of the ceramic clay and preservatives mixed with the pelleted feed (PG-D). Treatment diets were made by adding the appropriate treatment to the CON pelleted feed and mixed for 5 min in a small tumble mixer for 5 min. PowerGuard is a thermally processed ceramic particle that is micronized to a median particle size between 40 and 80 μm, with 90% of the particles being less than 100 to 150 μm. Briefly, dried and screened clay material is calcined in a natural gas-powered refractory dryer, which heats the material to a proprietary temperature for a precise amount of time. Following this step, the calcined material is micronized to optimize handling and surface area of the ceramic particles. PowerGuard composition is included in Table 1. Diet compositions are reported in Table 2. Pigs were 28 ± 3 d of age upon enrollment and initial body weights, 10.6 ± 1.25 kg, did not differ among treatments (*P* = 0.579). Pigs were fed their respective diets for a 20-d adaptation period followed by a 3-d total feed and fecal collection. At the end of the 3-d collection period pigs were immediately harvested and liver samples collected, flash frozen and stored in liquid nitrogen until further analysis of trace minerals and heavy minerals.

**Table 1.** Analyzed nutrient content of PowerGuard

Item	PowerGuard
DM, %	97.6
Ash, % DM	98.7
Ca, % DM	0.31
P, %DM	0.04
Mg, %DM	0.49
Na, %DM	0.08
K, %DM	0.59
S, %DM	0.22
Fe, %DM	3.28
Zn, %DM	0.12
Cu, %DM	<0.001
Mn, %DM	1.35

Minerals were analyzed by inductively coupled plasma optical emission spectrometry by Eurofins Scientific.

**Table 2.** Analyzed nutrient content of the treatment diets fed to weanling pigs

Item	Control^1^	PowerGuard^2^	PowerGuard-D^3^
DM, %	86.1	86.6	86.9
CP, %	23.3	23.3	23.3
TDN, %	86.9	86.9	86.9
Metabolizable Energy, Mcal/kg	3.9	3.9	3.9
Ash, %	6.1	5.6	5.5
NDF, %	15.8	15.6	15.9
ADF, %	5.1	5.3	5.2
Ca, %	1.0	1.0	1.0
P, %	0.7	0.7	0.7
Mg, %	0.2	0.2	0.2
Zn, ppm	3095	2878	2823
Cu, ppm	34.0	34.0	34.0
Thiamin, ppm	3.2	3.1	7.4
Retinol, IU/kg	3070	3090	3100

Ingredients: Ground corn, soybean meal, whey powder, porcine plasma, soybean oil, dicalcium phosphate, calcium carbonate, salt, zinc oxide, lysine, D,L-methionine, vitamin A acetate, vitamin D supplement, vitamin E supplement, vitamin k supplement, riboflavin, niacin supplement, calcium pantothenate, vitamin B12 supplement, iron proteinate, ferrous sulfate, zinc proteinate, zinc sulfate, manganese proteinate, manganese sulfate, copper proteinate, copper sulfate, sodium selenite, and calcium iodate.

Control diet with 0.25% of the DM containing a ceramic particle mixed into the pelleted feed (PG; MB Nutritional Sciences, Lubbock, TX, 79403).

Control diet with 0.3% of the DM containing a blend of preservatives and PG (PG-D; MB Nutritional Sciences, Lubbock, TX, 79403).

### Pig Feeding and Care

This study was conducted at the USDA Livestock Issues Research Unit Facility in New Deal, TX. Pigs were brought to the swine facility, weighed, and placed into individual elevated stainless-steel pens (1.2 × 0.6 m). This facility was temperature controlled with an average temperature of 28.5 °C. Pigs were offered ad libitum access to feed and water, where the automatic feeders were kept clean and refilled every other day or sooner as needed. When feeders were refilled, orts were first measured and the amount of feed given was measured, allowing for feed intake to be calculated.

### Sample Collection

Initial body weights were collected upon enrollment on day 0. On day 20, a 72-h total feed and fecal collection period began. Each pig and pen were observed every 15 min and fresh feces were collected when present by a trained individuals and stored in a fecal collection bag that stayed in a freezer in between collections. Feces were stored at −20 °C until analyzed of DM, CP, Ash, OM, ADF, NDF, zinc, copper, thiamin (vitamin B1), and retinol (vitamin A). Pigs were weighed for a final body weight and harvested on day 18 of the study via penetrative captive bolt immediately followed by exsanguination. Liver samples were collected immediately after harvest and frozen and stored in liquid nitrogen for subsequent mineral analysis.

### Sample Analyses

Fecal samples were analyzed for dry matter percentage using a two-stage drying process. First, the samples were dried at 55 °C in a forced air oven until the weight no longer changed, which was approximately 4 d for this study. Samples were ground using a coffee grinder and subsequently used for analytical measurements of nitrogen, ash, OM, ADF, NDF, zinc, copper, thiamin, and retinol concentrations. An additional 5-g sample was further dried at 100 °C for 24 h in a forced air oven to adjust to a 100% DM basis. Feed samples were dried and ground using a coffee grinder and analyzed similarly to the fecal samples. Fecal and feed samples were analyzed in duplicate. Organic matter and ash concentrations of fecal and feed samples were determined after the dry oxidation of samples at 550 °C in a furnace for 5 h (Cole Parmer, Vernon Hills, IL). Fecal and feed samples were analyzed for total nitrogen concentrations on a TruMac carbon, nitrogen, and sulfur autoanalyzer (Leco Corporation, St. Joseph, MI). The ADF and NDF concentrations in the fecal and feed samples were analyzed according to Van Soest (1991) using the Ankom Fiber Analyzer (Ankom^200^ Technology). Zinc and copper concentrations of fecal and feed samples were determined using inductively coupled plasma optical emission spectrometry (ICP-OES). Briefly, died samples were prepared in a MARS 6 microwave digestion system by CEM (Matthews, NC, USA). The method note for microwave digestion of dog feces was utilized as recommended by CEM. Samples were measured to 0.2 g in triplicate and digested with 10 mL of concentrated nitric acid (HNO_3_). Sample tubes were allowed to predigest for 15 min in a fume hood before being capped and digested at 200 °C and 800 psi for 15 min. Ramp time on the digestion system was 15 min with a total power of 900 to 1,050 W. Once cooled, samples were diluted 10 times with 2% HNO_3_ and analyzed for elemental contents using a ThermoFisher Scientific iCAP 7600 ICP-OES. Thiamin and total retinol concentrations were analyzed by a commercial laboratory using AOAC methods 942.23 and 974.29, respectively (Eurofins, Des Moines, IA). Apparent nutrient disappearance were calculated as follows: 100-((nutrient % in fecal sample × fecal output)/(nutrient % in feed sample × total DM feed intake)).

Liver samples were analyzed in duplicate by the Michigan State University Veterinary Diagnostic Laboratory (Lansing, MI 48910) for concentrations of retinol (vitamin A), iron, zinc, copper, manganese, cobalt, selenium, lead, mercury, arsenic, thallium, cadmium, and molybdenum. Retinol was quantified using ultra performance liquid chromatography and the minerals zinc and copper were quantified using ICP-OES.

### Statistical Analysis

All continuous, non-repeated data were analyzed using a restricted maximum-likelihood ANOVA with treatment supplementation included as the fixed effect and block as random effect using the MIXED procedure of SAS. Differences of *P* ≤ 0.05 were considered significant and a tendency was reported when 0.05 < *P* ≤ 0.10.

## RESULTS AND DISCUSSION

Supplementing clay in feed of multiple livestock species has shown health and performance benefits when added to a diet contaminated with mycotoxins or other biotoxins, but the concern with the high adsorption capacity of thermally processed clays is the potential for vitamins and trace minerals to be adsorbed on the clay surface or pores along with the targeted biotoxins (Stefanović et al., 2017; Li et al., 2018; Holanda & Kim, 2020). Various feed preservatives and antioxidants have been added to pig diets to detoxify or lessen the impact of mycotoxins such as deoxynivalenol (DON), which are difficult to adsorb. For example, when a sulfur group is transferred from a preservative like SMB onto DON, the DON becomes less toxic and exerts less detrimental health effects (Frobose et al., 2017). The concern with supplementing SMB to monogastric animals such as pigs is the potential reaction of SMB with stomach acid that can cause production of hydrogen sulfide gas, potentially leading to a decrease in health or performance. Further, feeding high sulfur in the diet can decrease the bioavailability of thiamin; therefore, thiamin is usually supplemented at greater concentrations in the diet with diets that are supplemented with SMB. The safety of feeding a supplement with relatively high levels of SMB to weanling pigs was evaluated in the current study and found to be safe to use as a feed additive, in agreement with Frobose and others in 2017.

Pig performance data including body weight, ADG, DMI, and feed to gain are all reported in Table 3. Initial body weight and final body weights did not differ among treatments (*P* ≥ 0.579). Further, there were no treatment differences for ADG, DMI or gain to feed ratio (*P* ≥ 0.701). Supplementation of an aluminosilicate montmorillonite clay to weanling pigs through finishing showed no overall differences in growth or digestibility, although it is interesting to note that final carcass weight and the carcass ratio were increased in the clay group supplemented at 0.8% of the diet when compared with the control (Kim et al., 2006). When ceramic particles were supplemented to pigs with or without a zearalenone contaminated diet, the ceramic particle had protective health effects as well as mediated aspects of the performance loss typically associated with mycotoxin contaminated feed (Jiang et al., 2010; Wang et al., 2012).

**Table 3.** Performance of piglets fed a control diet supplemented with either PowerGuard or PowerGuard-D

Item	Control^1,2^	PowerGuard	PowerGuard-D	SEM	*P =*
initial BW, kg	10.7	10.9	10.2	0.55	0.579
final BW, kg	22.5	22.9	22.1	1.10	0.859
ADG, kg/d	0.512	0.519	0.517	0.0298	0.986
DMI, kg/d	0.925	0.953	0.912	0.0417	0.788
Gain to Feed	0.552	0.541	0.565	0.0186	0.701

Treatments include the following: (1) Control, non-medicated commercial diet with no feed additive; (2) PowerGuard, basal diet with 0.25% of the DM containing a ceramic particle mixed with the pelleted feed (PG; MB Nutritional Sciences, Lubbock, TX, 79403); or (3) PowerGuard + preservatives, basal diet with 0.3% of the DM containing a blend of preservatives and PG with the pelleted feed (PG-D; MB Nutritional Sciences, Lubbock, TX, 79403).

Rows with differing superscripts indicate treatment differences with *P* < 0.05.

Nutrient digestibility data including DM, CP, OM, ADF, NDF, Ash, Cu, Zn, thiamin, and retinol are reported in Table 4. There were no treatment differences for DM, OM, ADF, NDF, or ash digestibility of the diets (*P* ≥ 0.312). Further, there were no treatment differences for copper, zinc, or retinol digestibility (*P* ≥ 0.298). There has been concern with clays processed specifically to increase their cation anion exchange capacity and the ability to bind micronutrients, such as trace minerals and some vitamins, and ultimately decrease bioavailability, but the current study did not reveal any changes in digestibility of vitamins or trace minerals when supplementing the diet with porous ceramic particles (Elliot et al., 2020).

**Table 4.** Apparent nutrient digestibility of pigs fed a control diet supplemented with either PowerGuard or PowerGuard-D

Item	Control^1,2^	PowerGuard	PowerGuard-D	SEM	*P*
Dry matter, %	92.1	92.6	91.4	0.71	0.501
Crude protein, %	91.9	92.7	91.6	0.79	0.646
Organic matter, %	92.8	93.3	92.2	0.63	0.481
Acid detergent fiber, %	67.1	71.9	65.9	3.05	0.363
Neutral detergent fiber, %	82.2	83.6	81.4	1.74	0.668
Ash, %	80.9	80.1	76.4	2.15	0.312
Zinc, %	68.7	71.5	63.9	3.29	0.298
Copper, %	61.9	68.5	60.5	4.25	0.391
Thiamin (vitamin B1), %	83.4^a^	83.3^a^	92.8^b^	2.28	0.018
Retinol (vitamin A), %^3^	98.7	98.7	98.6	0.12	0.621

Treatments include the following: (1) Control, non-medicated commercial diet with no feed additive; (2) PowerGuard, basal diet with 0.25% of the DM containing a ceramic particle mixed with the pelleted feed (PG; MB Nutritional Sciences, Lubbock, TX, 79403); or (3) PowerGuard + preservatives, basal diet with 0.3% of the DM containing a blend of preservatives and PG with the pelleted feed (PG-D; MB Nutritional Sciences, Lubbock, TX, 79403).

Rows with differing superscripts indicate treatment differences with *P* < 0.05.

Estimated because fecal excretion was below the limit of detection (60 IU/100g), so fecal output was calculated as limit of detection × fecal DM output.

Montmorillonite clays are organized by layers of mineral sheets stacked upon one another, and are connected by a combination of interlayer cations, van der Waals forces, H-bonds, or electrostatic forces (Carrara Di Gregorio et al., 2014; Uddin, F., 2018; Unuabonah et al., 2018). The quality and quantity of the interlayer cations changes the cation exchange capacity (CEC) of the clay (Emmerich, 2009). The potential for clays to adsorb minerals and interfere with digestibility of nutrients increases with the CEC and the specific cations present. Due to the thermal processing applied to the ceramic particle used in the current study as well as the extremely low CEC, the data from the current study support the idea that PG and PG-D do not adsorb or interfere with micro- or macromineral digestion.

In a study supplementing weanling pigs a sodium bentonite clay at 1% of the diet contaminated with aflatoxin-B1 (AFB1), the uncontaminated clay group did not have different CP digestibility when compared with any other treatment; however, the DM digestibility was decreased by 1.4%. Further, it was determined that the clay supplemented treatments had decreased mineral absorption, including phosphorous, magnesium, iron, and zinc. The authors suggested that increased dietary mineral content from the clay increased concentrations of dietary minerals over the nutrient requirements and may have interfered with normal absorption and retention rates of those minerals (Schell et al., 1993). Other literature supplementing aluminosilicate clays to pigs decreased the DM digestibility only at high inclusion rates of 0.75%, whereas the 0.25%, and the 0.5% inclusion rates did not affect any diet digestibility. No difference in any inclusion level was reported for energy, nitrogen, or phosphorous digestibility (Thacker, 2003). When the same clay product was supplemented at an inclusion rate of 0.5%, no differences were reported in DM or phosphorous digestibility but a tendency to increase nitrogen digestibility and ADG was determined when compared with the control group (Chen et al., 2005). Contrastingly, supplementation of a silicate clay to growing pig diets at 0.5% and 1.0% of the diet revealed increased DM, nitrogen, calcium, and phosphorous digestibility for both inclusion rates, as well as increased measures of performance and health (Li and Kim, 2013).

Liver mineral data are reported in Table 5 and include dry liver sample weight, retinol, iron, zinc, copper, manganese, cobalt, selenium, lead, mercury, arsenic, thallium, cadmium, and molybdenum. There was no treatment difference in dry weight of the liver samples (*P* = 0.502). There were no treatment differences for liver concentrations of retinol, iron, zinc, copper, manganese, cobalt, selenium, arsenic, thallium, cadmium, or molybdenum (*P* ≥ 0.509). These data are consistent with the lack of a difference in apparent nutrient digestibility; however, the short duration of feeding should be noted as a limitation of these data. There were tendencies for treatment differences in liver concentrations of both lead and mercury (*P* ≤ 0.066), where the PG-D treatment group tended to have greater liver concentrations of both lead and mercury when compared with both the CON and PG treatments. These data suggest that the preservatives used in the formulation of PG-D may have contained slightly elevated concentrations of these two heavy metals.

**Table 5.** Hepatic nutrient concentration of pigs fed a control diet supplemented with either PowerGuard or PowerGuard-D

Item	Control^1,2^	PowerGuard^®^	PowerGuard-D^®^	SEM	*P*
Dry weight, %	30.4	30.9	29.8	0.60	0.502
Retinol, ppm	112.5	106.4	113.5	11.02	0.886
Iron, ppm	156.8	169.2	135.0	34.68	0.784
Zinc, ppm	2538	2380	2632	150.9	0.509
Copper, ppm	22.3	21.4	19.1	2.54	0.655
Manganese, ppm	10.1	10.6	9.9	0.47	0.532
Cobalt, ppm^3^	<0.04	<0.04	<0.04	…	…
Selenium, ppm	2.07	2.09	2.1	0.08	0.681
Lead, ppm	0.10	0.11	0.16	0.017	0.063
Mercury, ppm	0.41	0.40	0.43	0.007	0.066
Arsenic, ppm^3^	<0.08	<0.08	<0.08	…	…
Thallium, ppm^3^	<0.08	<0.08	<0.08	…	…
Cadmium, ppm^3^	<0.08	<0.08	<0.08	…	…
Molybdenum, ppm	2.54	2.25	2.60	0.294	0.679

Treatments include the following: (1) Control, non-medicated commercial diet with no feed additive; (2) PowerGuard, basal diet with 0.25% of the DM containing a ceramic particle mixed with the pelleted feed (PG; MB Nutritional Sciences, Lubbock, TX, 79403); or (3) PowerGuard + preservatives, basal diet with 0.3% of the DM containing a blend of preservatives and PG with the pelleted feed (PG-D; MB Nutritional Sciences, Lubbock, TX, 79403).

Rows with differing superscripts indicate treatment differences with *P* < 0.05.

Reported as below the limit of detection.

There was a treatment difference for thiamin digestibility (*P* = 0.018), where the PG-D treatment group had increased thiamin digestibility compared to both the CON and PG treatments. It is important to note that excess sulfur in a diet can cause thiamin deficiency in pigs, where excess dietary sulfur intake can interfere with cytochrome oxidase, brain metabolism, and bind to or make thiamin otherwise unavailable for metabolic processes (Hough et al., 2014). Due to this potential dietary interaction, the PG-D diet was supplemented with approximately an additional 4.35 ppm thiamin to account for the potential interaction between the additional sulfur in the diet. Therefore, the increase in thiamin digestibility for the PG-D treatment may be due to the increased inclusion rate of thiamin in the diet. Dietary inclusion of SMB in DON contaminated diets has shown beneficial effects on health and performance of pigs. When DON is present in pig feed, it is rapidly absorbed and metabolized, leading to adverse impacts on both health and performance. When DON contaminated feed is supplemented with SMB, the sulfur group combines with DON to form 10-sulfonate adduct, or DON-S, which is not as deleterious to pigs. In models of both high and low concentrations of DON contamination, supplementation of SMB in the diets showed benefits on ADG, BW, ADFI, as well as decreased DON availability within the feed (Frobose et al., 2015; Shawk et al., 2018).

The objective of this study was to determine the impact of supplementing a montmorillonite-based ceramic particle with or without preservatives on the performance and apparent nutrient digestibility of weanling pigs. Ultimately, the ability of ceramic particles to impact performance depends on the health status or biotoxin exposure of the animal, the classification of ceramic particle and the dietary inclusion rate. In the current study, there were no impacts on performance or apparent nutrient digestibility among treatments in these healthy weaned pigs, especially the micronutrients.

## Notes

Mention of trade names or commercial products in this article is solely for the purpose of providing specific information and does not imply recommendation or endorsement by the U.S. Department of Agriculture.

The U.S. Department of Agriculture (USDA) prohibits discrimination in all its programs and activities on the basis of race, color, national origin, age, disability, and where applicable, sex, marital status, familial status, parental status, religion, sexual orientation, genetic information, political beliefs, reprisal, or because all or part of an individual’s income is derived from any public assistance program. (Not all prohibited bases apply to all programs.) Persons with disabilities who require alternative means for communication of program information (Braille, large print, audiotape, etc.) should contact USDA’s TARGET Center at (202) 720-2600 (voice and TDD). To file a complaint of discrimination, write to USDA, Director, Office of Civil Rights, 1400 Independence Avenue, S.W., Washington, D.C. 20250-9410, or call (800) 795-3272 (voice) or (202) 720-6382 (TDD). USDA is an equal opportunity provider and employer.
